# Aboveground net primary productivity not CO_2_ exchange remain stable under three timing of extreme drought in a semi-arid steppe

**DOI:** 10.1371/journal.pone.0214418

**Published:** 2019-03-26

**Authors:** Hui Zhang, Hua Yu, Chaoting Zhou, Haitao Zhao, Xiaoqing Qian

**Affiliations:** 1 College of Bioscience and Biotechnology, Yangzhou University, Yangzhou, China; 2 College of Life Sciences, University of the Chinese Academy of Sciences, Beijing, China; 3 Department of Foreign Languages, University of the Chinese Academy of Sciences, Beijing, China; 4 College of Environmental Science and Engineering, Yangzhou University, Yangzhou, China; Tennessee State University, UNITED STATES

## Abstract

Precipitation patterns are expected to change in the semi-arid region within the next decades, with projected increasing in extreme drought events. Meanwhile, the timing of extreme drought also shows great uncertainty, suggesting that the timing of drought, especially during growing season, may subsequently impose stronger stress on ecosystem functions than drought itself. However, how the timing of extreme drought will impact on community productivity and carbon cycle is still not clear. In this study, three timing of extreme drought (a consecutive 30-day period without precipitation event) experiments were set up separately in early-, mid- and late-growing season in a temperate steppe in Inner Mongolia since 2013. The data, including soil water content (SWC), soil temperature (ST) chlorophyll fluorescence parameter (*F*_*v*_*/F*_*m*_), ecosystem respiration (Re), gross primary productivity (GPP), net ecosystem carbon absorption (NEE) and aboveground net primary productivity (ANPP) were collected in growing season (from May to September) of 2016. In this study, extreme drought significantly decreased SWC during the drought treatment but not for the whole growing season. Extreme drought decreased maximum quantum efficiency of plant photosystem II (*F*_*v*_*/F*_*m*_) under “optimum” value (0.75~0.85) of two dominant species (*Leymus chinensis* and *Stipa grandis*). While ANPP kept stable under extreme drought treatments due to the different responses of two dominant species, which brought a compensating effect in relative abundance and biomass. In addition, only early-growing season drought significantly decreased the average Re (*P* < 0.01) and GPP (*P* < 0.01) and depressed net CO_2_ uptake (*P* < 0.01) than mid- and late-growing season drought. ST and SWC influenced the changes of GPP directly and indirectly through photosynthetic ability of the dominant species by path analysis. Our results indicated that the timing of drought should be considered in carbon cycle models to accurately estimate carbon exchange and productivity of semi-arid grasslands in the context of changing climate.

## Introduction

General atmospheric circulation models predict changes in precipitation patterns, for anthropogenic activity increase atmospheric CO_2_ concentrations and temperature [1–3]. These changes may lead to more severe or more frequent extreme drought events in the future, at both local and global regions [4, 5]. The drought events can result in more stress on water availability and therefore affect plant and ecosystem functions, depending on local historical weather, plant phenological stage [6–8] and the involved species identities and abundance [9]. Importantly, the timing of arising drought would play an equally critical role as the severity of drought itself, or even a more vital role on ecosystem functions than drought alone, probably by affecting plant phenological and physiological responses [10–13]. Especially, in semi-arid area, the stressful drought events would play a determinant role in controlling the development of ecosystem productivity [7, 12] and carbon (C) exchange [14–17].

Many ecosystem functions, such as aboveground net primary productivity (ANPP) and net ecosystem CO_2_ exchange [NEE: The difference between C sequestration through gross primary productivity (GPP) and release through ecosystem respiration (Re)], are the most frequently considered variables in extreme climate studies [18–20]. Drought has been well documented negatively to affect ecosystem C uptake across the biomes [21–23]. For example, drought leads to ecosystem net C loss by declining species photosynthesis [24]. In contrast, drought also can increase maximum canopy carbon uptake by facilitating shoot to root ratio and therefore increasing primary production [25]. The divergent responses of carbon exchange to extreme drought depend on ecosystem and the duration of drought [4]. In addition, some empirical model studies have suggested that extreme drought, which is defined as consecutive days without rainfall, can decrease net C sequestration due to the more sensitive response of GPP than Re [24, 26] constrained by soil water content. Moreover, soil temperature may be another factor in controlling ecosystem C uptake, but sometimes the relationship between soil temperature and Re is decoupled under drought condition [14]. Consequently, ecosystem C exchange is assumed to be strongly determined by soil water availability [27]. By now, manipulated extreme drought experiments have applied to water reduction treatments [26, 28–31], while the effect of timing of extreme drought is always ignored. Moreover, not all drought will equally influence C exchange and productivity. Therefore, it is necessary to consider more diverse drought events, like timing of extreme drought, in manipulative experiments which could shed light on the direction and magnitude of carbon exchange in responses to extreme drought events [32].

In addition, at species level, the drought influences plant physiological and phenological responses by limit of soil water availability. The negative effects feedback to ecosystem functions, such as ecosystem productivity in the semi-arid grassland [6, 7, 11, 33]. Unfortunately, only a few studies have paid attention to the effects of the timing of drought to ecosystems [6, 7, 11, 12, 33]. Additionally, the previous studies have focused more on the effects of drought on individual plants, but ignored the overall connection between species and ecosystem functions [6, 7].

Specially, a study on early stage of the growing season, when plant begins to store energy to prepare for the following stages of growing and flowering, showed plant’s negative physiological response to drought [34]. In contrast, on late stage of the growing season, plant is close to senescence and may not be sensitive to response to drought, and therefore drought at this stage will result in few effects on productivity [6]. Therefore, we assume that drought in early-growing season induces more substantial effects on plant physiology, productivity and carbon exchange than drought in other stages of the growing season. and how different response or relationship between the plant and ecosystem.

Dominant species, which contribute for the most important parts of community net primary productivity, play a key role in response of ecosystem functions and services toward climate change, especially to drought [25, 35, 36]. Particularly, chlorophyll fluorescence, as one of plant ecophysiological parameters, controls ecosystem CO_2_ exchange and productivity [37]. Additionally, as a part of first stage of photoreaction, chlorophyll fluorescence shows the detailed information of photosystem II (PS II) with less damage [38]. The ratio of varied fluorescence (*F*_*v*_) to maximum fluorescence (*F*_*m*_) is calculated as the maximum quantum efficiency of photosystem II. The decreased maximum quantum efficiency (*F*_*v*_*/F*_*m*_) indicates drought stress, which decreases plant photosynthetic capacity while increases photoprotection when drought occurs [39]. The research showed that the negative effect of drought on *F*_*v*_*/F*_*m*_ can induce carbon fixation in a grassland in central Europe [25]. Unfortunately, it is still unclear with regards to the effects of the timing of extreme drought on *F*_*v*_*/F*_*m*_ and whether the responses of *F*_*v*_*/F*_*m*_ determine CO_2_ exchange and productivity.

Here, we conducted a field experiment of extreme drought events to investigate the potential influence of the timing of drought on plant physiology, ecosystem carbon exchange and aboveground net primary productivity (ANPP) in a semi-arid temperate steppe in Inner Mongolia, China, which covers *ca*. 4.1× 10^7^ hm^2^ and accounting for 10.5% of the total area of Chinese grassland. The drought events at this region have increased over the past 50 years, with annual and seasonal drought events increased by 37% [40], especially drought in the early growing season [41]. Given precipitation drives the development of productivity and distributions of plant life forms [42], this typical temperate steppe of Inner Mongolia is an optimal location to investigate the generality of ecosystem responses to projected shifts of extreme precipitation patterns. By now, many studies have paid attention to how drought events, including extreme drought, affect ecosystem functions [25, 43], but less attention to the impacts of the timing of drought. It is highly needed to investigate how the timing of drought affects ecosystem functions. Therefore, the specific questions we addressed in this study included: (1) how does *F*_*v*_*/F*_*m*_, ANPP and ecosystem CO_2_ exchange respond to the timing of extreme drought? (2) how do both biotic and abiotic factors drive the response of ANPP and CO_2_ exchange to the timing of extreme drought?

## Materials and methods

### Site description

The study site locates at the Maodeng pasture (44°11'N, 116°27'E), Inner Mongolia Autonomous region, China. The soil is chestnut (Calcic Chernozem according to ISSS Working Group RB, 1998). This region is characterized by a temperate continental climate with a mean annual temperature of -1.4°C and a mean annual precipitation of 350 mm [21, 44] based at the past 60 years. Mean growing season precipitation is 280 mm, accounting for 80% of total annual precipitation, of which 75% is considered as ecologically effective precipitation (EP, recorded daily precipitation > 3 mm during the growing season) [45]. The community at this steppe comprises a mixture of annual and perennial species (including grasses and forbs). The xeric rhizomatous grass (*Leymus chinensis*), needle grass (*Stipa grandis*) and *Cleistogenes squarrosa* are the dominant species [21]. *Artemisia frigida*, *Potentilla acaulis*, and *Chenopodium glaucum* constitute a large proportion of the total number of individual plants, but their biomass only accounts for a small fraction of the total (<10%).

### Experimental design

The treatment of extreme drought started in 2013. A randomized block experiment with 4 replicates for each treatment was designed to test the effects of timing of extreme drought on performance of plant photosynthetic traits and ecosystem CO_2_ exchange. Three patterns in terms of different timing of extreme drought were set up across the growing season: early-growing season drought (Early-D, June-July), mid-growing season drought (Mid-D, July-August), late-growing season drought (Late-D, August-September). The timing of drought corresponds to the vegetative phase, reproductive phase and senescence phase of the dominant species (*L*. *chinensis* and *S*. *grandis* in particular). An ambient treatment was treated as control. Based on a ~60 years’ data provided by the local meteorology station, a consecutive 30 days without effective ecological precipitation is defined as extreme drought within fitting Gumbel I distribution model [13]. Rain-out shelters (4.5 m × 6 m of height 3 m, details see Hao et al., 2018) were used to exclude rain from the treatment plots (2 m ×2 m) during the treatment.

### Soil water status and soil temperature

Volumetric soil water content (SWC) was measured at 10 cm by a time domain reflectrometry (TDR 300, Spectrum Technologies, Inc. CST, USA) that was inserted in the soil vertically. Soil temperature (ST) at a depth of 10 cm below the surface was monitored by using a thermometer set in the center of each plot (Model TL-883, Tonglixing technology Co., Ltd., China).

### Chlorophyll fluorescence measurement

Two dominant species, *L*. *chinensis* and *S*. *grandis*, widely distributed in this region, were selected to measure chlorophyll fluorescence through a pulse-amplitude-modulated photosynthesis yield analyzer with Dual-PAM (Walz, Germany). Predawn fluorescence values were measured on non-senescent mature leaves at the pre-, during-, post- extreme drought treatment. The following parameters were based on Maxwell & Johnson (2000):

Fv=Fm−F0 1

*F*_*v*_ *is* variable fluorescence from dark- adapted leaves. *F*_*0*_ is minimal fluorescence and *F*_*m*_ is maximal fluorescence. The ratio of *F*_*v*_ *to F*_*m*_ is maximum quantum efficiency of photosystem II.

### Ecosystem CO_2_ exchange measurement

Ecosystem CO2 exchange, including NEE and Re, was measured by a transparent chamber attached to an infrared gas analyzer (LI-840A, LI-COR Inc., Lincoln, NE, USA). A 50 cm length × 50 cm width × 10 cm depth steel frame was installed to a 7 cm depth of soil in each plot before the onset of the treatments. The NEE and Re were measured in the morning (09:00–11:30) on sunny days to ensure data comparability throughout the growing season and to avoid physiological stress brought by high air temperature at noon. For the details of NEE and Re measurement see Hao et al., (2018). Briefly, CO_2_ concentration within chamber was recorded every second lasting for 2 minutes. After measuring NEE, the chamber was immediately lifted up and was covered with a black lightproof cloth to estimate Re. GPP was calculated through the difference between NEE and Re. The data for the first and last 10s were deleted when calculated ecosystem CO_2_ exchange.

### Aboveground net primary productivity (ANPP)

For 16 plots, after recording the abundance of each species, aboveground net primary productivity was estimated by clipping aboveground living plants in a 0.5×0.5 m^2^ quadrat at the end of the experiment. Clipped plants were immediately brought back to laboratory. These clipped samples were oven-dried at 65°C to a constant weight, which was recorded subsequently. In addition, all plants were grouped into three categories: *L*. *chinensis*, *S*. *grandis* and others (species only accounting for a small percentage).

### Data analysis

All statistical analysis was performed on R 3.2.5 (R Development Core Team, 2016) with the package of “agricolae” and “stats”. The effects of extreme drought treatment on soil water content, soil temperature, chlorophyll a fluorescence parameters and CO_2_ exchange were compared through analysis of variance (ANOVA) over the whole growing season. Duncan’s Multiple Range Test was employed to test the differences among the average values of NEE, Re, GPP and soil volumetric water content, when the significance level reached 0.05. To address the specific effects of extreme drought, student’s t-test analysis on *F*_*v*_*/F*_*m*,_ SWC, NEE, Re and GPP were performed to test the differences between extreme drought and ambient treatments at the same time, i.e. pre-treatment (Pre-T), during-treatment (Dur-T) and post-treatment (Post-T). Before the significance test, the normality of error terms was evaluated according to the *Kolmogorov-Smirnov* test, and homoscedasticity was evaluated through the *Levene* test for equality of variances. We performed path analysis to measure the effects of the abiotic factors (SWC and ST) and biotic factor (sum the *F*_*v*_*/F*_*m*_ of *L*. *chinensis* and *S*. *grandis* as the ecosystem photosynthetic performance) on GPP by using the standardization of multiple linear regression models (SPSS Amos 25, SPSS Inc., Chicago, USA). Specially, the adequacy of the model was tested using chi-square (χ^2^) tests, standardized root mean square residual (SRMR) index, root-mean-square error of approximation (RMSE) index and goodness-of-fit index (CFI).

## Results

### Growing season precipitation and changes in soil volumetric water content

**Fig 1:**
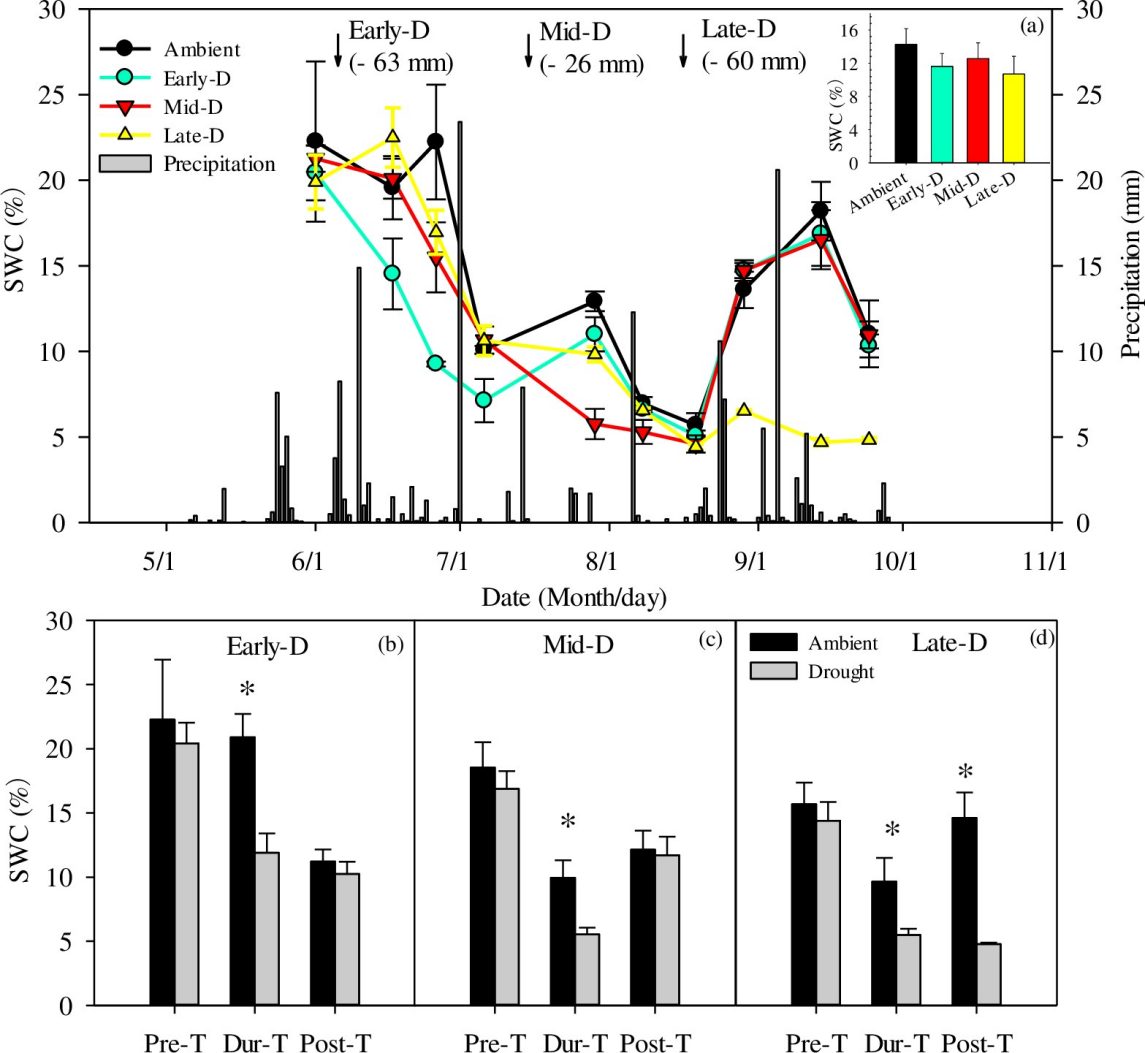
(a) Distribution of daily precipitation (mm) and dynamics in daily mean soil water content (%) at 10 cm depth during the growing season in 2016. The inset plot represents the average soil water content in 2016. (b, c, d) The mean soil water content detected at pre- (Pre-T), during- (Dur-T) and post- (Post-T) treatment. Early-D, Mid-D and Late-D mean early-, mid- and late-growing season extreme drought, respectively. The values in parentheses mean the reduced rainfall compared to the control of the same period. *: Significant differences between extreme drought (D) and ambient treatment at *P*≤0.05.

The study year was a drought year when compared with the historical averages. In 2016 growing season, the ambient precipitation was 165 mm, while the long-term mean growing season precipitation of this site was 280 mm (1952–2013). The precipitation amounts of early- and late-growing season extreme drought were close to the mean value of the corresponding period of the ~60 years’ record, except the treatment of mid-growing season extreme drought, whose precipitation was less than half of the mean value in the ~60 years’ record. During the extreme drought treatment of Early-D, Mid-D and Late-D, the excluded precipitation was 63, 26 and 60 mm respectively in 2016 (Fig 1A).

All treatments decreased soil water content (SWC) during the treatment significantly (Fig 1B, 1C and 1D), which was 43~46% lower than SWC in the ambient plot. After extreme drought treatment, SWC in Early-D, and Mid-D plots showed a better recovery than that under the ambient conditions after drought treatment. However, SWC in Late-D did not achieve a good recovery due to fewer precipitation input in the post-treatment period. Across the whole growing season, extreme drought decreased the average soil water content of all three treatments compared with the ambient treatment, but it did not reach the significantly statistic level (*P* = 0.59). The declination of the mean soil water content in Early-D, Mid-D and Late-D were by 19%, 12%, and 25%, compared with ambient treatment. Early-D and Late-D treatments showed more effects on mean soil water content than Mid-D treatment did.

### Response of chlorophyll fluorescence to extreme drought

**Fig 2:**
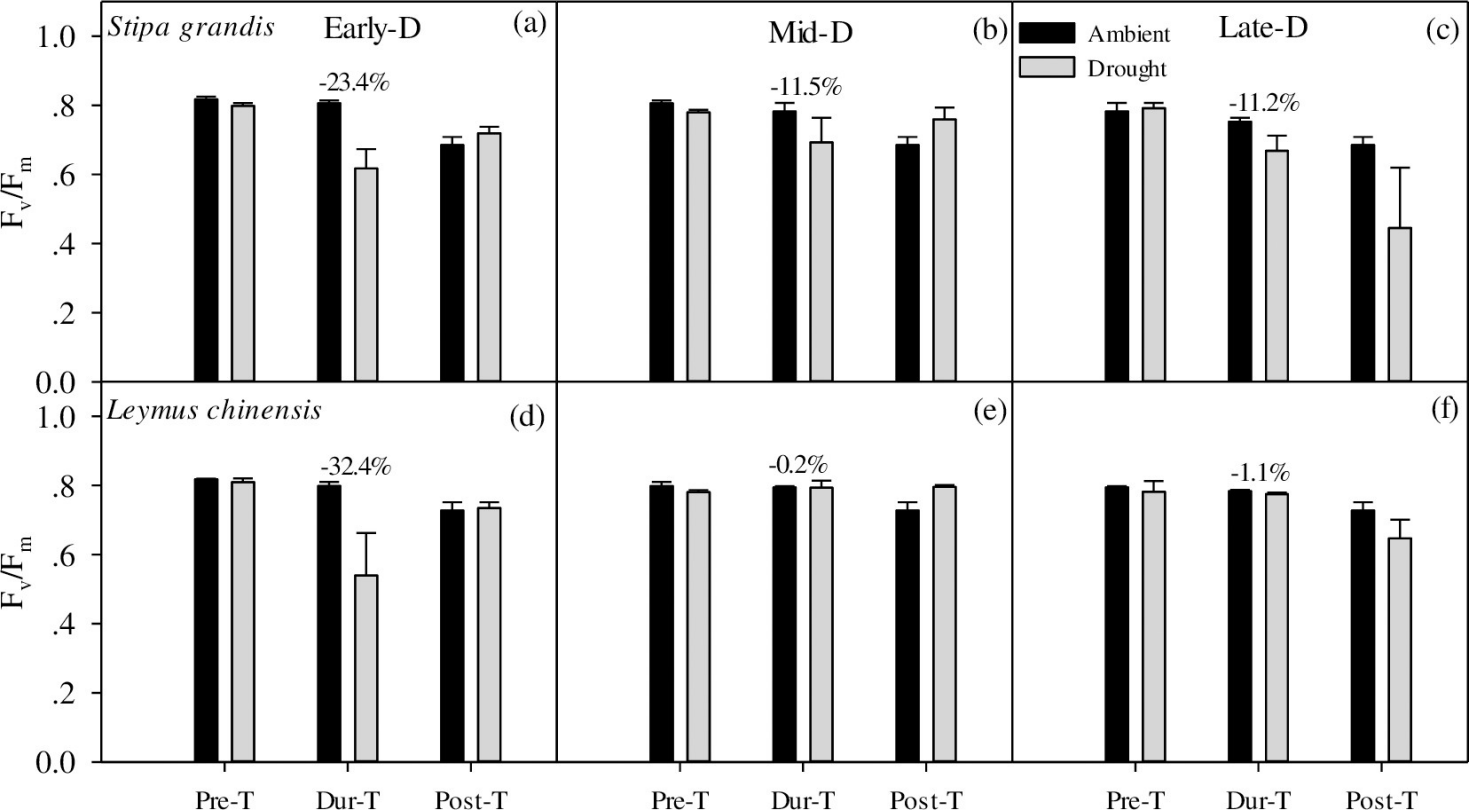
**Variations in the average of *F***_***v***_***/F***_***m***_ **of (a-c) S*tipa grandis* and (d-f) *Leymus chinensis* during pre-, during- and post- treatment of three drought treatment.** The percentage at the top of the bar represents decreased values of treatments. Data are means±se.

For the two dominant species of *L*. *chinensis* and *S*. *grandi*, maximum quantum efficiency (*F*_*v*_*/F*_*m*_) varied in a range of 0.54~0.82. Although there was no statistical effect of extreme drought on *F*_*v*_*/F*_*m*_, extreme drought decreased plant chlorophyll fluorescence parameter during the drought treatment, which fell below the effective value (0.75~0.85, Fig 2). These negative effects were particularly distinct in Early-D treatment for both *L*. *chinensis* and *S*. *grandis*. Particularly, in terms of chlorophyll fluorescence parameter, the negative response to extreme drought shown by *L*. *chinensis* showed was more distinct than that by *S*. *grandis*, with the decrease of 32% in *L*. *chinensis* and 23% in *S*. *grandis* during the treatment in Early-D treatment. However, chlorophyll fluorescence of *S*. *grandis* showed to be more sensitive to drought than that of *L*. *chinensis*, and the former decreased by 12% and 11% in Mid-D and Late-D respectively. After the drought treatment, *F*_*v*_*/F*_*m*_ of two species in Early-D and Mid-D treatments, not in Late-D treatment, however, showed a physiological recovery with an effective physiological value.

### Response of CO_2_ exchange to extreme drought

**Fig 3:**
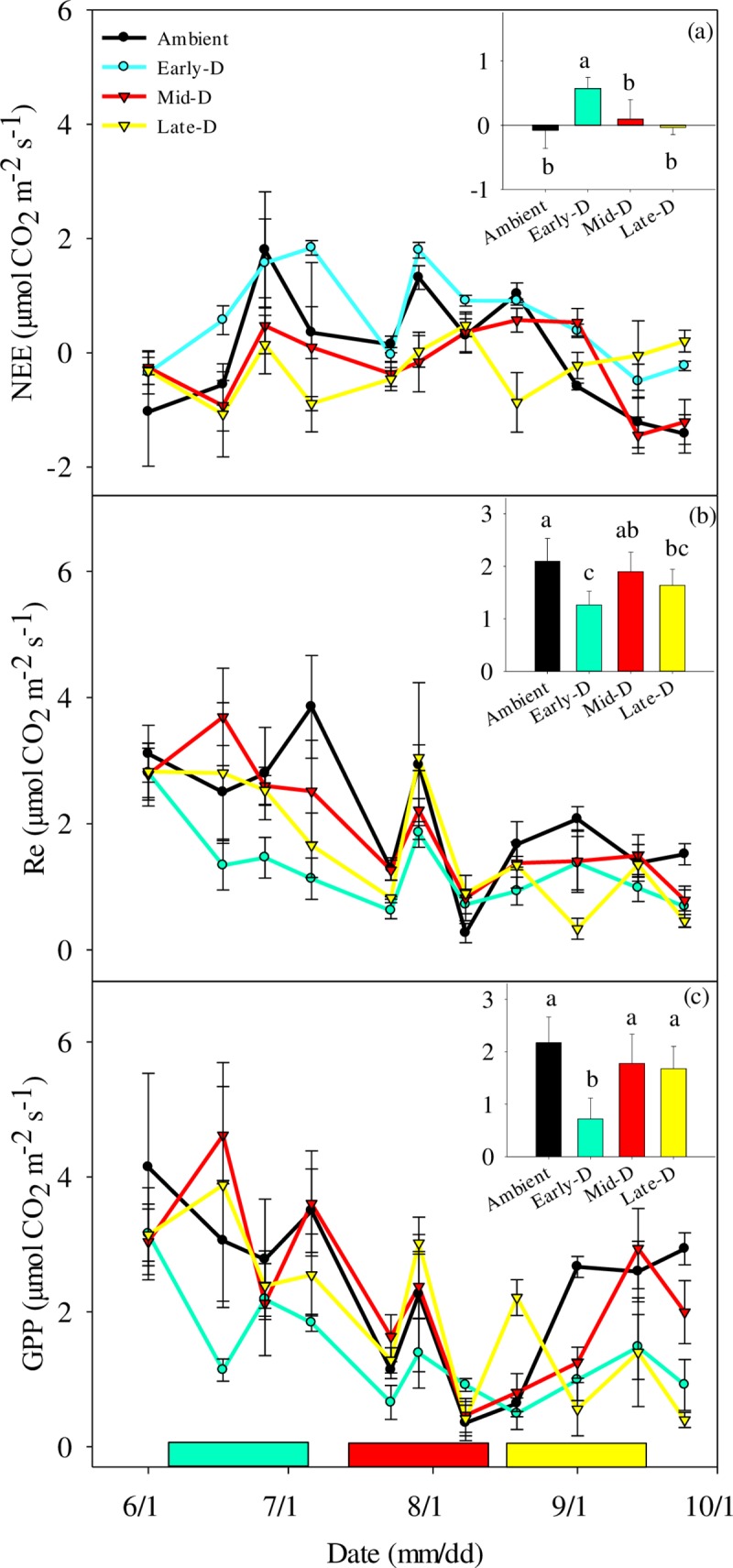
**Seasonal variations in (a) net ecosystem CO**_**2**_ **exchange (NEE), (b) ecosystem respiration (Re) and (c) gross primary productivity (GPP) of the treatments and ambient plots in 2016’s growing season.** Each inset plot represents the average NEE, Re and GPP in 2016. The colored rectangles in (c) represent corresponding time of the three drought treatments. Data are means±se.

Across the whole growing season, extreme drought events significantly influenced the means of NEE (*P* < 0.01), Re (*P* < 0.01) and GPP (*P* < 0.01) (Fig 3). The negative response to drought shown in Early-D was more distinct than those shown in Mid-D and Late-D. Early-D significantly increased seasonal NEE (negative values mean net CO_2_ uptake from the atmosphere) and released average CO_2_ (0.56 μmol CO_2_ m^-2^ s^-1^) to the atmosphere, while the ambient plots were carbon-neutral over the growing season (NEE~0).

**Fig 4:**
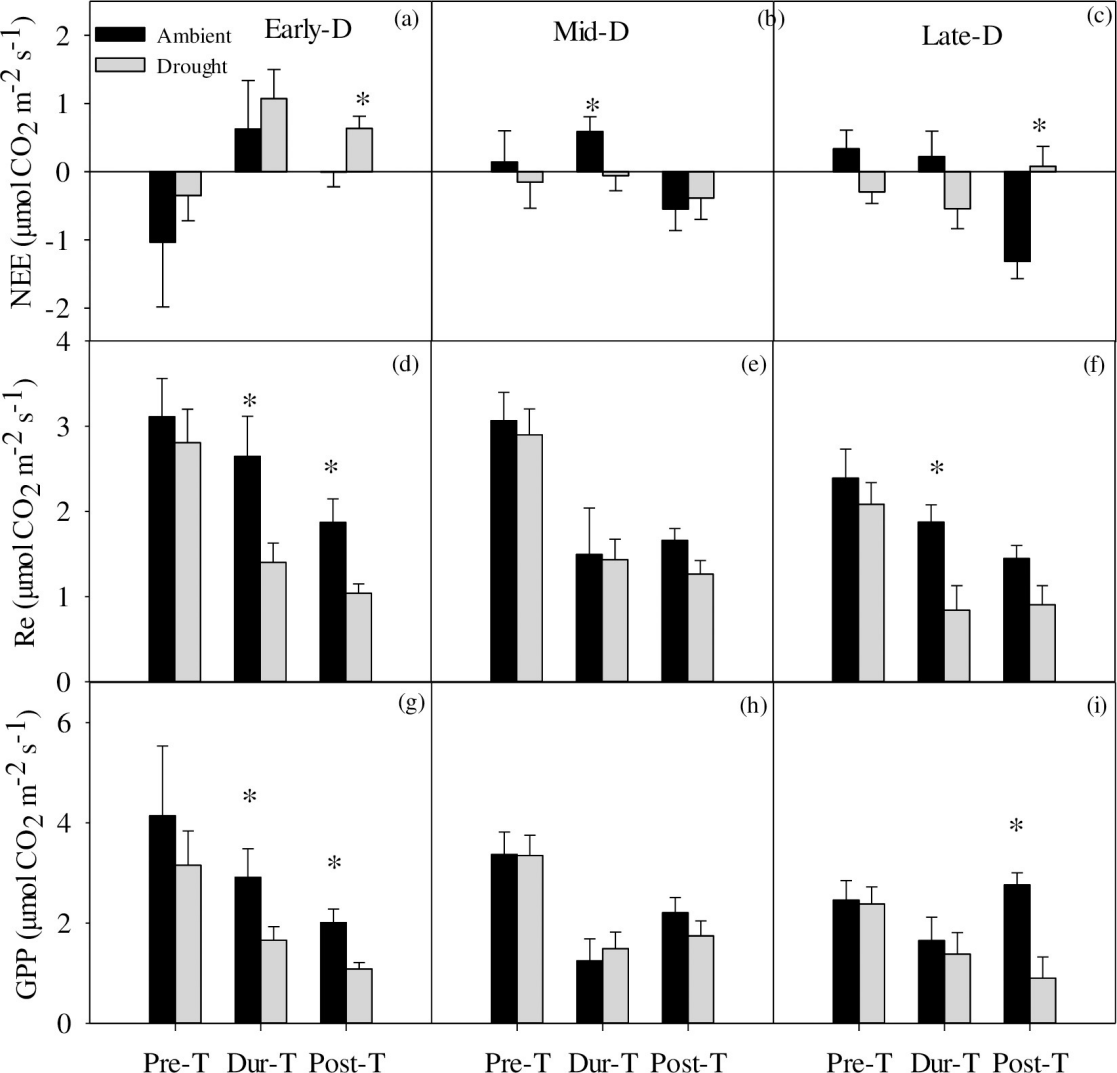
**Variations in (a-c) average NEE, (d-e) Re and (g-i) GPP of pre-, during- and post-treatment of three drought treatment.** Data are means±se.

In the perspectives of three drought stages, only Mid-D significantly increased CO_2_ uptake during the treatment (Fig 4A), while Early-D and Late-D significantly increased CO_2_ release at the post-treatment (Fig 4A and 4C). In Early-D and Late-D, extreme drought significantly decreased Re by 47% and 57% respectively (Fig 4D and 4F) during the treatment. These negative effects lasted till the post time in Early-D treatment. Extreme drought showed no significant effect on Re of Mid-D (Fig 4E). GPP showed a decreasing trend during drought treatments and lasted till the post-treatment in Early-D and Late-D. (Fig 4G and 4I). Drought in Early-D and Late-D significantly reduced GPP by 46% and 68% compared with the ambient treatment after drought treatment.

### Response of ANPP to extreme drought

**Fig 5:**
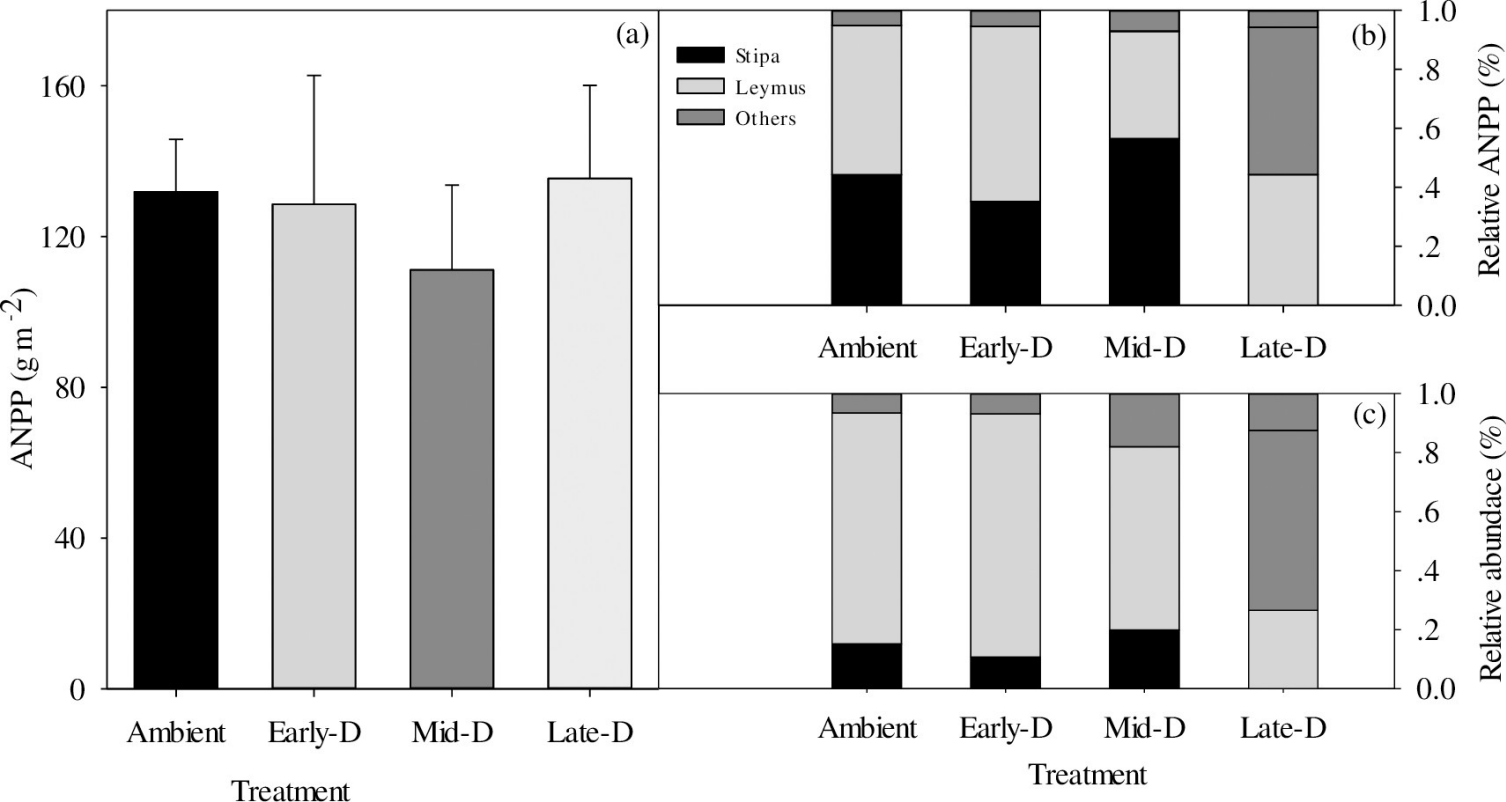
(a) Effects of timing of extreme drought on aboveground net primary productivity (ANPP, g m^-2^) in 2016. The (b) relative ANPP and (c) relative abundance of species in each treatment. Stipa, Leymus and others represent *Stipa grandis*, *Leymus chinensis* and other species, which were only occupied a small percentage in the typical steppe. Data are means±se.

At the community level, extreme drought showed no significant effect on ANPP (*P* = 0.24; Fig 5A). However, all ANPP of these three drought treatments in 2016 were lower than those in the long-term mean ANPP (186 g m^-2^). The xeric rhizomatous grass *(L*. *chinensis)* and the needle grass (*S*. *grandis*) took up to 90% of the ANPP in all plots. In addition, the variation in relative ANPP of these two dominant species was observed in all of the drought treatments (Fig 5B). ANPP of *S*. *grandis* increased by 28% in Mid-D treatment, while it remained stable in Late-D. However, ANPP of *L*. *chinensis* increased by 17% in Early-D compared with the ambient treatment. Furthermore, the relatively abundance of *L*. *chinensis* in Early-D increased by 6% compared with ambient treatment (Fig 5C). While the relatively abundance of *S*. *grandis* in Mid-D and Late-D increased by 31% and 75% respectively, the relative abundance of *L*. *chinensis* decreased almost by 20% both in Mid-D and Late-D.

### Abiotic and biotic factors control over the response of CO_2_

**Fig 6:**
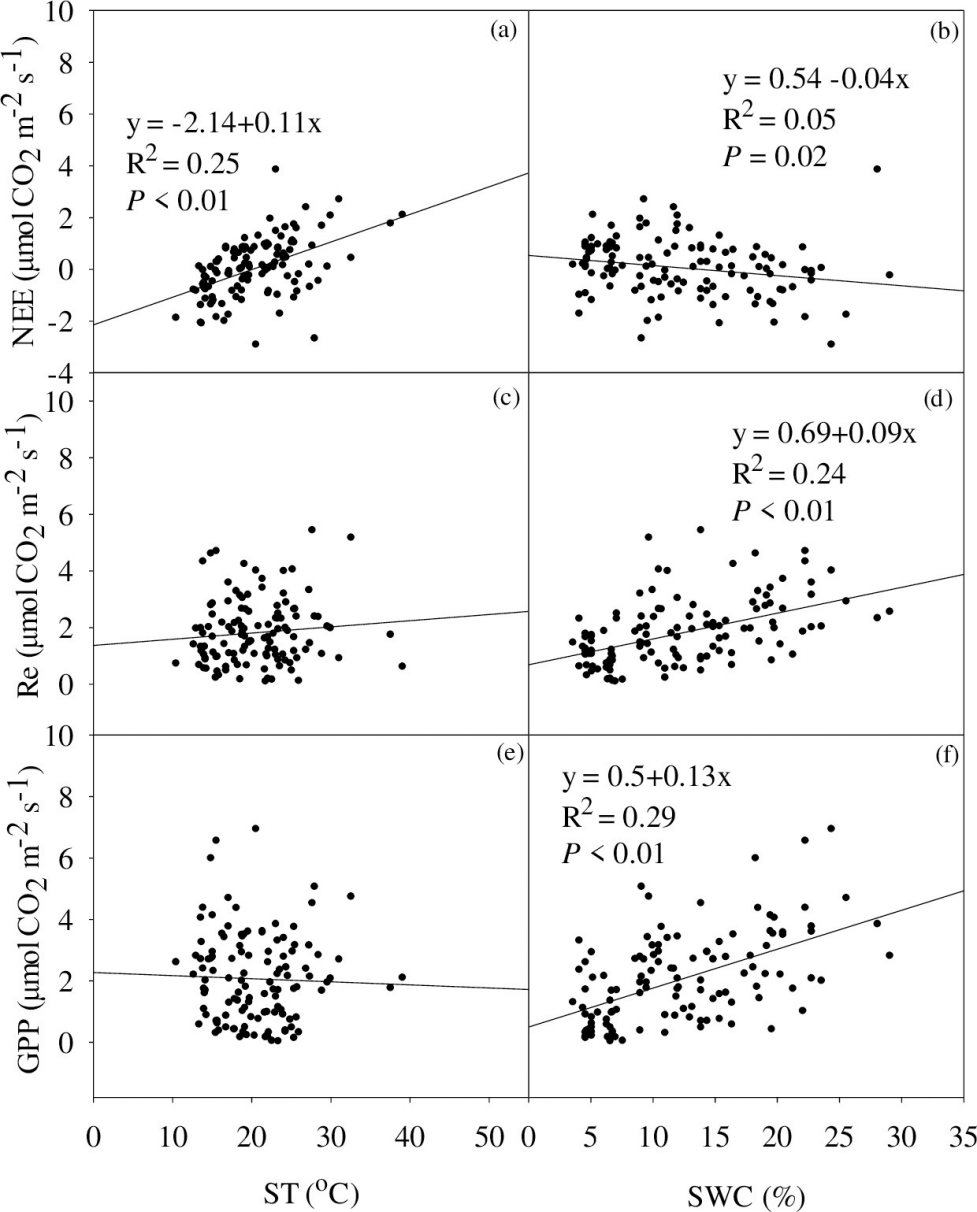
**Relationships between soil temperature (ST,°C) and (a) NEE (c) Re (e) GPP, and soil water content (SWC, %) and (b) NEE (d) Re (f) GPP**.

Net ecosystem CO_2_ exchange and its two components (GPP and Re) had stronger relationships with soil water content (SWC) than with soil temperature. (ST, Fig 6). A weakly negative linear relationship between NEE and SWC was observed (R^2^ = 0.1), while there were significantly positive linear relationships between GPP, Re and SWC (R^2^ = 0.29 for GPP, R^2^ = 0.24 for Re). However, only NEE, rather than GPP and Re, had a positive linear relationship with soil temperature (R^2^ = 0.25).

**Fig 7:**
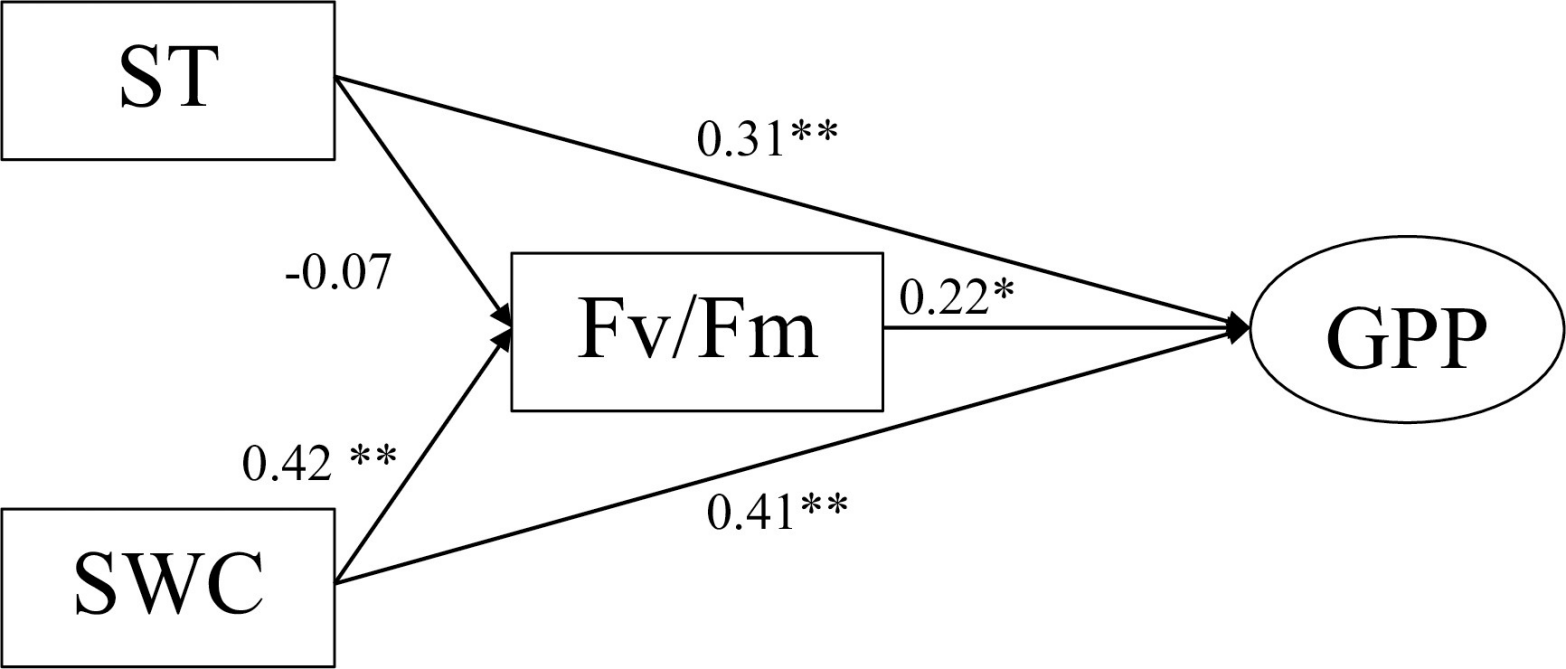
Path diagram illustrates the effects of abiotic factors (ST and SWC) and biotic factor (sum the *F*_*v*_*/F*_*m*_ of *L*. *chinensis* and *S*. *grandis* as the ecosystem photosynthetic performance) on gross primary productivity (GPP). χ^2^ = 0.78, *df* = 1, *P* = 0.38, RMSEA = 0.00, AGFI = 0.92, CFI = 1.00, Significance codes mean: * *P* < 0.10, ** *P* < 0.05 and *** *P* < 0.001.

Path analysis showed that soil water content (standardized coefficient = 0.41, *P* = 0.001) and soil temperature (standardized coefficient = 0.31, *P* = 0.008) had a positive effect on GPP in this steppe ecosystem (Fig 7). At the community level, soil water content (standardized coefficient = 0.42, *P* = 0.002) had a positive effect on maximum quantum efficiency of plant photosystem II, while soil temperature (standardized coefficient = -0.07, *P* = 0.5) had a negative effect on maximum quantum efficiency of plant photosystem II. Ecosystem photosynthetic performance had a positive effect on GPP (standardized coefficient = 0.22, *P* = 0.08). These results suggested that soil water content under extreme drought events affected plant physiological activity and consequently influenced ecosystem productivity as well.

## Discussion

### Response of ANPP to extreme drought

In our research, we had exposed a semi-arid steppe to extreme drought during three stages of growing season (early-, mid- and late-growing season) since 2013. These three drought periods corresponded with vegetative phase, reproductive phase and senescence phase of the dominant species of *L*. *chinensis*, perennial herb with developed rhizomes, and *S*. *grandis*, perennial herb with dense clusters. We collected the data in 2016 and found that all these three extreme drought treatments had no significant effect on ANPP in this steppe, which indicated that the community productivity kept stable when facing extreme drought. Additionally, we found that the relative percentage of two dominant species’ ANPP and abundance were altered in dominant species. Specifically, in Early-D, *L*. *chinensis* increased while *S*. *grandis* decreased in the relative ANPP and abundance. However, in Mid-D and Late-D, *S*. *grandis* increased while *L*. *chinensis* decreased in the relative ANPP and abundance. *L*. *chinensis* and *S*. *grandis* presented different physiological response to these three drought treatments. Particularly, Early-D had a large negative effect on chlorophyll fluorescence of two dominant species. *L*. *chinensis* showed stronger negative response to drought than *S*. *grandis* in Early-D (Fig 2A and 2D). However, in Mid-D and Late-D, *S*. *grandis* showed stronger negative response to drought than *L*. *chinensis*. The reason can be partly explained by the vegetative stage of *S*. *grandis* was lasting longer than *L*. *chinensis*, which began to reproduce at the mid-growing season and its growth was less influenced by drought, while *S*. *grandis* began to reproduce at the late-growing season [27, 46, 47]. Another reason for these different responses may lie in the different function types of these two species and their different life forms [48]. Meanwhile, the resource allocation, such as leaf mass ratio, was different when faced with drought stress [48, 49]. These altered conditions on relative ANPP, abundance and chlorophyll fluorescence implied that these two dominant species had different strategies to respond to the timing of extreme drought, and they may have kept the stability of ecosystem when they were exposed to the timing of extreme drought.

Importantly, it should be noted that the study year of 2016 was a drought year with only 165 mm precipitation during the growing season, which was much lower than the average annual growing season precipitation of 280 mm. Bai et al. (2004) found that ANPP was positively line with precipitation amount. Lower precipitation may attenuate the difference of ANPP between extreme drought treatments and ambient plots. Therefore, no significant response of ANPP was observed when facing drought treatments. Another primary reason may be attributed to the high ecosystem resistance in this steppe. The steppe experienced chronic fluctuations under the climate change, especially in precipitation patterns with a decreasing tendency [2, 3]. The plants had adapted to these changes and have applied relevant strategies to respond to these fluctuations and stresses. Grasslands showed high resistance in face of stressful conditions due to physiological down-regulation and limitations of nutrition, such as lack of soil nitrogen [15, 25, 50]. Studies in this steppe have highlighted the stability of ecosystem response to simulated drought perturbation [15, 42, 51].

### Response of CO_2_ exchanges to extreme drought

CO_2_ exchange showed more variables than ANPP. Specially, the negative effects on NEE, Re and GPP was more distinct in the Early-D than those in Mid-D and Late-D (Fig 3). A consecutive 30-day period without precipitation event occurring at early-growing season caused a significant decrease in net CO_2_ uptake and switched the ecosystem from a CO_2_ sink to a CO_2_ source (Fig 3). In addition, the different response of ecosystem CO_2_ exchange to extreme drought were different at the different stages of growing season. NEE and Re under the extreme drought treatment showed significant response during the drought treatment while GPP did so only after the treatment (Fig 4), especially in early- and late-growing season drought which excluded more rain (~60 mm) than mid-growing season drought (26 mm). Previous studies using eddy-covariance technique have found that CO_2_ exchange is sensitive to drought and is negatively correlated with annual precipitation [23, 52, 53]. Similarly, in field experiments, drought decreased net ecosystem CO_2_ exchange by reduced soil moisture [21], stimulated root mortality [54, 55], soil microbial activity [56] and consequently suppressed plant assimilation [27]. In addition, previous studies suggested GPP was more sensitive to drought than Re resulted in depression of NEE [18, 27, 57]. Drought can cause the declination of ecosystem CO_2_ sink by decreased leaf area index and water use efficiency [44]. During the drought treatment, CO_2_ exchange significantly showed negative response to drought and even continued until the end of the experiment. In general, extreme drought may dramatically affect ecosystem CO_2_ exchange and then ecosystem functions and services.

### The influence of timing of extreme drought on ANPP and CO_2_ exchange

Recently, studies paid attention to the timing and then the intensity of extreme drought. Researches have found that at the beginning of the growing season, precipitation plays a crucial role in plant growth [54]. Another study showed that precipitation from January to July was the prior driver for inducing community biomass fluctuated [42]. In our study, we found ANPP kept stability in 2016 after 4 years of extreme drought treatments. Interestingly, the effect of drought on ANPP was independent on its occurring time. This temperate steppe showed stronger stability than our expectation. Additionally, “drought memory” of plants can also partly explain the stability of ANPP to extreme drought, because this experiment had been operated since 2013 [39]. However, some documents suggested that the timing of drought played an important role in affecting ecological processes [55, 58, 59]. For example, early-growing season drought can decrease the rate of growth of ponderosa pine seedlings [33] and species richness in a semi-natural grassland in Switzerland [7]. Both early- and mid-growing season drought decreased ANPP in a prairie [12]. In our research, the differences among early-, mid- and late-growing drought in growing season did not seem to perform distinctly in ANPP, but distinctly in GPP, Re, NEE and chlorophyll fluorescence, especially in different treatment period. It was worth noting that the early-growing season drought decreased Re and GPP, then decreased net carbon uptake. After the adjustment of plant physiology like chlorophyll fluorescence, the negative effects during the treatments recessed to a beneficial level (similar values to ambient plot) in the post time of treatments except late-growing season drought.

### The relationship between CO_2_ exchange and biotic and abiotic factors

In the semi-arid temperate steppe, soil moisture availability was a vital factor in driving carbon exchange. Thus, it was necessary to investigate the relationship between CO_2_ exchange and soil water content caused by extreme drought. Our results found that the CO_2_ exchange was significantly sensitive and was correlated with soil water content. It was closely consistent with the results from some of the previous studies [15, 52]. Similarly, both GPP and Re linearly descended with the reducing soil water content [27] in drought ecosystem. And ecosystem carbon exchange had been proved to be strongly depended on soil water content. Moreover, soil temperature was also correlated with the NEE, ascending soil temperature increased CO_2_ release [21] in a steppe.

The performance of PS II, an important photosynthesis component, influenced the plant assimilation under the climate change, and then affected the ecosystem CO_2_ exchange and productivity [25, 60]. We found that *F*_*v*_*/F*_*m*_ fell below the “optimum” value (0.75~0.85) [35] under the extreme drought treatments, especially during the drought treatment. *L*. *chinensis* was more sensitive than *S*. *grandis* in early-growing season drought. The negative response was also found in a grassland system in central Europe [61]. The drought decreased *F*_*v*_*/F*_*m*_ obviously in a desert ecosystem. Furthermore, the negative physiological responses matched the changes of NEE in early-growing season drought, which changed ecosystem from carbon sink to carbon source (Fig 3A).

Since the abundance and biomass of *L*. *chinensis* and S. *grandis* reached up to 90% (Fig 5), we took the sum *F*_*v*_*/F*_*m*_ of the two dominant species as the photosynthetic performance of the whole community [62] to investigate the effect of drought on the ecosystem’s GPP. As the primary process of carbon cycling, efficiency of photosystem II determined the CO_2_ assimilation process. In our path analysis, we found soil water content directly affected photosynthetic performance of community, and consequently affected soil water content (path coefficient = 0.41) and soil temperature (path coefficient = 0.31) directly attributed a lot to the change of GPP. As a result, the change of photosynthetic performance of the community affected fluctuation of GPP (path coefficient = 0.22). In contrast, remote sensing data indicated that the decrease in the plant photosynthetic performance did not result in a declination in GPP in a temperate grassland [62]. Overall, our research exhibited the importance of biotic and abiotic factors on GPP under seasonal timing of extreme drought, directly or indirectly by soil water content, soil temperature and photosynthetic performance of dominant spices.

## Conclusions

In our study, extreme drought events were manipulated in order to study the responses of plant physiological parameter, ecosystem carbon exchange and ecosystem productivity. Our results showed that, compared with mid-growing season drought, the extreme drought in early- and late-growing season demonstrated a bigger influence on Re during treatment (Dur-T) and GPP in the recovery time (Post-T). Early-growing season drought decreased Re and GPP and increased net carbon release to a larger degree while extreme drought in mid-growing season showed less effects on carbon exchange. However, CO_2_ exchange was not consistent with ANPP, which kept stable under the timing of extreme drought. Soil water content and soil temperature affected GPP directly and even indirectly by photosynthetic performance (chlorophyll fluorescence) of dominant species. Our results suggest that, to better estimate the carbon exchange and productivity of semi-arid grasslands in the context of changing environment, timing of droughtshould be considered in climate change models.
